# Effects of a game-based learning ıntervention on self-reported learning strategies and professional attitudes in kinesiophobia management education: a parallel-group randomized controlled trial

**DOI:** 10.1186/s12909-026-09782-8

**Published:** 2026-06-25

**Authors:** Neslihan Altuntaş Yılmaz, İrem Caner

**Affiliations:** 1 Nezahat Keleşoğlu Faculty of Health Sciences, Physiotherapy and Rehabilitation, Necmettin Erbakan University, Meram, Konya, Türkiye; 2 Necmettin Erbakan University, Institute of Health Sciences, Konya, Türkiye

**Keywords:** Game-based learning, Physiotherapy education, Learning strategies, Professional attitudes, Randomized controlled trial

## Abstract

Innovative educational strategies are increasingly needed in physiotherapy education to support students’ learning and professional development. Game-based learning (GBL) is an interactive, student-centered approach that may enhance engagement and active participation. This randomized controlled trial investigated the effects of a structured GBL intervention on self-reported learning strategies and professional attitudes in kinesiophobia management education among physiotherapy students. Ninety-six final-year physiotherapy students were randomly assigned to an intervention group (*n* = 48) or a control group (*n* = 48). The intervention group participated in a five-week GBL program consisting of theoretical sessions, case-based simulations, and gamified escape-room activities, while the control group followed the standard curriculum. The primary outcome was self-reported learning strategy scores assessed using the Learning Styles Scale for Health Sciences Students. Professional attitudes, assessed using the Attitude Scale Towards the Physiotherapy Profession, were considered a secondary outcome. Data were analyzed using t-tests and mixed ANOVA (α = 0.05). Ninety-four students completed the study. Compared with the control group, the intervention group demonstrated significantly higher post-intervention visual (*p* = 0.001) and kinesthetic (*p* = 0.034) learning strategy scores. Significant group × time interactions were observed for visual (η^2^p = 0.118, *p* < 0.001), tactile (η^2^p = 0.074, *p* = 0.004), and auditory (η^2^p = 0.230, *p* < 0.001) domains. No significant group × time interaction was observed for kinesthetic learning strategies (*p* = 1.000). Professional attitude scores did not change significantly in either group (*p* > 0.05). A structured GBL intervention may serve as a useful complementary educational approach for enhancing selected self-reported learning strategy domains in physiotherapy education. However, no short-term effects were observed on professional attitudes. Future studies should include objective outcomes and longer follow-up. NCT07334899 (Retrospectively registered on December 31, 2025).

**Trial registration**

NCT07334899 (Retrospectively registered on December 31, 2025).

**Supplementary Information:**

The online version contains supplementary material available at https://doi.org/10.1186/s12909-026-09782-8.

## Introduction

Learning is fundamentally defined as a relatively permanent change in an individual’s behavior and cognitive processes resulting from experience. Traditionally, the concept of learning style has been used to describe a consistent pattern of cognitive, affective, and physiological characteristics that determine how learners perceive and interact with their environment [10]. However, contemporary educational research suggests that learning preferences should not be viewed as fixed learner characteristics and that students may benefit from exposure to multiple instructional approaches rather than teaching methods tailored to a single learning strategy [27, 32]. The ability to employ multiple learning approaches simultaneously allows individuals to analyze situations systematically, relate observations to theoretical frameworks, and actively regulate their own learning processes [29].

In the context of health education, the integration of game-based elements dates back to the 1980 s [3], emerging as a strategy to enhance clinical skills and engagement [19]. Educational games typically incorporate rules, objectives, problem-solving, critical thinking, and competition to create interactive environments [13]. While traditional teaching methods remain prevalent, evidence suggests that active learning strategies like game-based learning (GBL) are increasingly incorporated into health sciences education [9] and can produce superior learning outcomes compared to conventional instruction [42]. These characteristics make GBL particularly relevant for physiotherapy education, where active participation, clinical reasoning, and problem-solving skills are essential components of professional training [33].

Modern educational models increasingly emphasize the integration of diverse learning strategies to create inclusive environments. Visual learning facilitates comprehension through graphics and representations [38], while auditory learning reinforces knowledge through verbal interaction [36]. Kinesthetic learning is particularly relevant in applied disciplines like physiotherapy, as it promotes learning through movement and hands-on experience [18]. While these learning preferences are frequently discussed in educational research, they should be interpreted as flexible and context-dependent tendencies rather than fixed learner categories [27]. Furthermore, problem-based learning enhances critical thinking [39], social learning approaches foster collaboration [17], and technology-supported platforms enrich the educational process (Choi-Lundberg 2023). Integrating these strategies helps address diverse learning preferences [26]. Although many innovative and game-based approaches have been investigated in physiotherapy- and rehabilitation-related contexts, including studies examining attention, physical performance, and biopsychosocial outcomes [4, 31], evidence regarding their educational application in physiotherapy training remains limited.

Despite growing evidence supporting GBL in health professions education, little is known about its effectiveness in teaching kinesiophobia management to physiotherapy students. Furthermore, no randomized controlled trials have specifically examined whether a structured GBL intervention can influence self-reported learning strategies and professional attitudes in this context.

With the rapid advancement of technology, GBL has emerged as an innovative method to make learning more motivating and engaging [43]. These environments offer substantial benefits by utilizing the inherent motivation associated with gameplay to achieve educational objectives [46]. Research indicates that GBL not only supports skill development but also promotes productive and meaningful engagement with learning content [44].

A critical area for physiotherapy education is the management of kinesiophobia, a frequently encountered condition in patients undergoing rehabilitation [15]. Defined as an excessive and irrational fear of physical activity due to concerns about re-injury, kinesiophobia is prevalent in 50% to 70% of individuals with chronic pain [11]. This condition can severely limit participation in daily life activities, making education on its management a vital competency for physiotherapy students [22].

Kinesiophobia develops through direct aversive experiences, such as pain or trauma, as well as social learning processes. According to the fear-avoidance model, the perceived threat of pain depends on individual and contextual factors. While avoidance behavior may serve as a short-term protective response, it paradoxically contributes to the persistence of pain and reduced quality of life in the long term [23]. Given the rising importance of innovative teaching strategies and the clinical relevance of kinesiophobia, the aim of this study was to investigate the effects of a game-based learning intervention on self-reported learning strategies and professional attitudes among physiotherapy students. We hypothesized that students participating in the game-based learning intervention would demonstrate greater improvements in self-reported learning strategy scores and professional attitudes than students receiving the standard curriculum alone.

## Materials and methods

### Study design

This study was conducted as a parallel-group, randomized controlled, pretest–posttest trial. The study was designed, conducted, and reported in accordance with the Consolidated Standards of Reporting Trials (CONSORT) 2010 Statement. A completed CONSORT checklist is provided as a supplementary file. The primary outcome was self-reported learning strategy scores assessed using the Learning Styles Scale for Health Sciences Students, whereas professional attitudes were considered secondary outcomes.

### Participants

The sample size was determined based on effect sizes reported in previous randomized controlled game-based learning studies, which have shown moderate effects on learning outcomes in health education [6]. The sample size calculation was based on the primary outcome measure (self-reported learning strategy scores). A priori power analysis using G*Power 3.1 indicated that, assuming a type I error of 5% (α = 0.05), 80% power, and a medium effect size (d = 0.60), a total of 90 participants (45 per group) would be sufficient for an independent samples t-test.

**Fig. 1:**
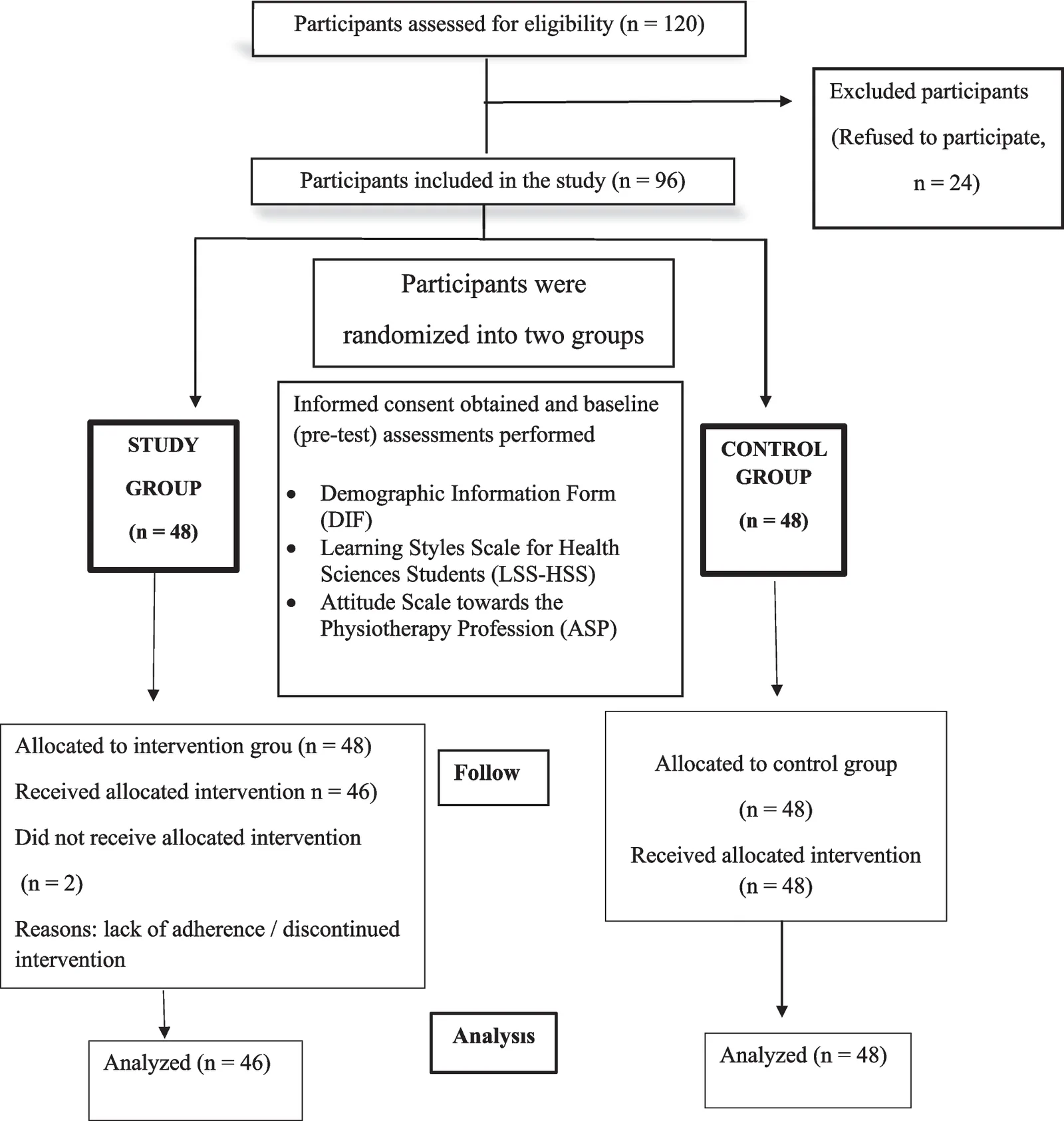
Study flow diagram of participant recruitment, randomization, follow-up, and analysis

Of the 120 final-year Physiotherapy and Rehabilitation students assessed for eligibility, 24 declined to participate. The remaining 96 participants were randomly allocated to either the intervention (*n* = 48) or control (*n* = 48) group using computer-generated randomization (Fig. 1). The study was conducted between February and May 2025 at Necmettin Erbakan University, Nezahat Keleşoğlu Faculty of Health Sciences.

All participants provided written informed consent, and the study was conducted in accordance with the Declaration of Helsinki. This study was reviewed and approved by the Necmettin Erbakan University Scientific Research Ethics Committee for Health Sciences (decision no: 2025/932, meeting no: 51, dated January 08, 2025). This study was retrospectively registered at ClinicalTrials.gov (Identifier: NCT07334899) on December 31, 2025.

Inclusion criteria were: (1) being enrolled in the final year of the Physiotherapy and Rehabilitation program, (2) voluntary participation, and (3) completion of all baseline assessments. Exclusion criteria were: (1) inability to understand Turkish, (2) difficulty completing self-report questionnaires, and (3) withdrawal during the study period.

### Randomization and group allocation

After providing written informed consent and completing baseline assessments, participants were randomly assigned to either the intervention or control group using a computer-generated randomization sequence. The allocation sequence was generated by a researcher not involved in outcome assessment. To ensure allocation concealment, group assignments were placed in sequentially numbered, opaque, sealed envelopes. Following completion of the baseline assessments, each participant was assigned to a group according to the next envelope in the sequence. This procedure was implemented to minimize selection bias and maintain allocation concealment throughout the enrollment process.

### Blinding

Two researchers were involved in the study. Due to the nature of the intervention, participants were aware of their group allocation (not blinded). Because of the educational nature of the intervention, participant blinding was not feasible. However, to ensure objectivity of the measurements, the researcher responsible for data evaluation was blinded to group allocation. The other researcher conducted the intervention procedures and was aware of the group assignments.

### Data collection ınstruments

Data were collected using the Demographic Information Form, the Attitude Scale towards the Physiotherapy Profession (ASP), and the Learning Styles Scale for Health Sciences Students (LSS-HSS).

The Demographic Information Form included variables such as age, gender, grade point average, encounter with kinesiophobic patients during education, working status, university preference order, willingness in choosing the profession, and liking the profession.

The Attitude Scale towards the Physiotherapy Profession (ASP) was used to evaluate professional attitudes. Developed by Turhan [40], it is a five-point Likert-type scale consisting of 35 items. Except for two items (26th and 30th), 33 items are scored from 1 (strongly disagree) to 5 (strongly agree), while two items are reverse scored. The total score ranges from 35 to 175, with higher scores indicating more positive attitudes toward the physiotherapy profession. The scale has three subdimensions (professional satisfaction, professional qualifications, and general concerns about the profession). Cronbach’s alpha coefficient of the scale is 0.977, and Cronbach’s alpha values for the subdimensions are 0.966, 0.974, and 0.957, respectively.

The Learning Styles Scale for Health Sciences Students (LSS-HSS) was used to assess participants’ self-reported learning strategy preferences [30]. The scale evaluates four learning strategy domains (visual, tactile, auditory, and kinesthetic). It is a self-report, five-point Likert-type instrument. The relatively higher overlap observed in kinesthetic items is attributed to its conceptual relationship with tactile and visual learning characteristics.

### Intervention

**Fig. 2:**
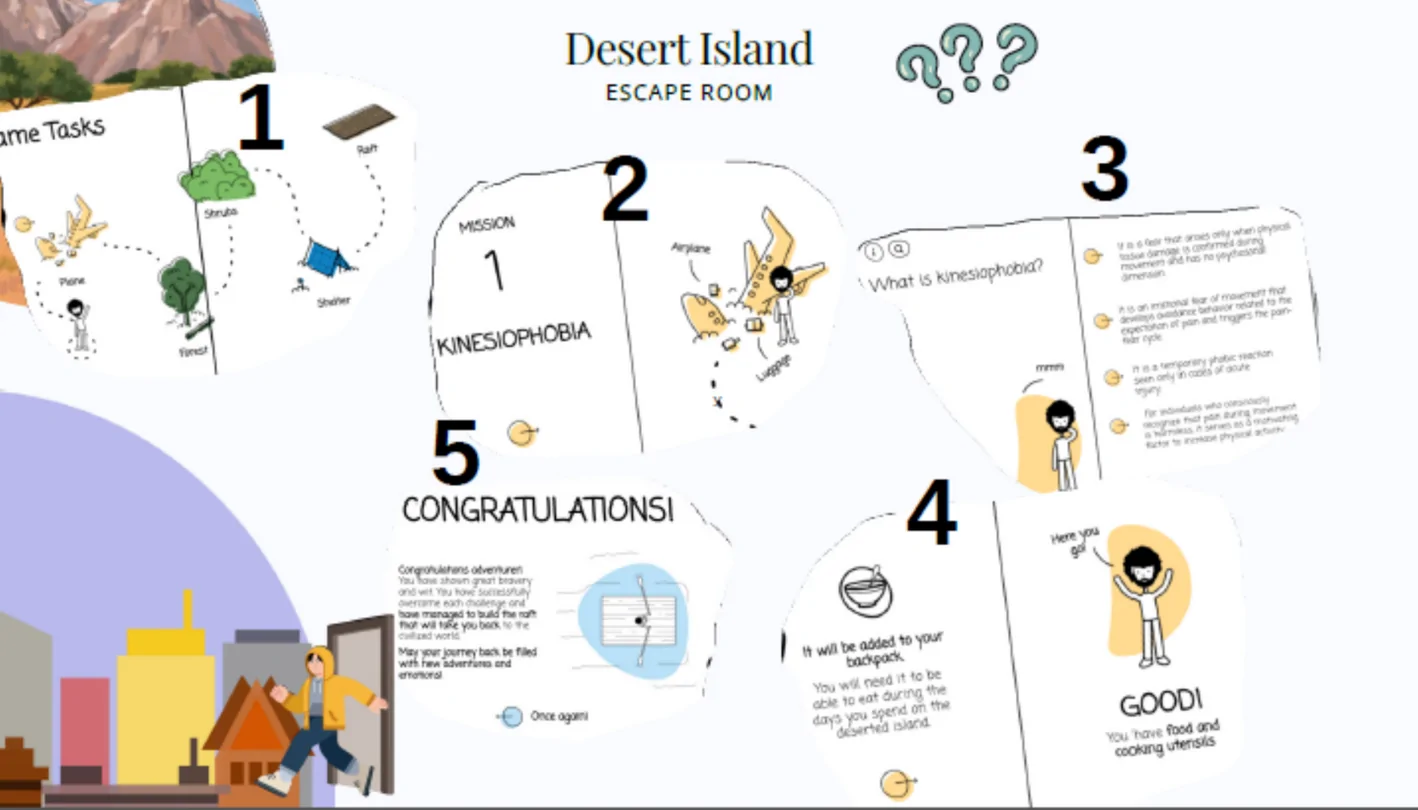
Escape room–based learning activity

Both groups continued their standard curriculum throughout the study. In addition, the intervention group participated in a five-week game-based learning program on approaches to patients with kinesiophobia. The program included weekly 50 min theoretical sessions, case simulations, and gamified “Escape Room”–themed quizzes [28, 37] (Fig. 2). The intervention was delivered by a faculty member experienced in physiotherapy education. Each weekly session consisted of a theoretical component followed by case-based discussions and gamified learning activities. During the case simulations, students were presented with patient scenarios involving different levels of kinesiophobia, functional limitations, and psychosocial characteristics. Students were encouraged to identify potential risk factors, select appropriate assessment methods, and develop individualized management strategies.

The training focused on developing assessment and treatment plans through patient scenarios with varying psychological and physical profiles. Gamified quizzes prepared using the Genially platform reinforced learning on key topics such as the definition and assessment of kinesiophobia, appropriate management strategies, behaviors to avoid, home recommendations, and the role of patient relatives. The Escape Room activities required students to work collaboratively to solve sequential clinical tasks and answer case-related questions in order to progress through the activity. Immediate feedback was provided following each task to reinforce learning objectives and facilitate discussion of correct and incorrect responses.

Example quiz questions included:

1. What is kinesiophobia?
2. For which patient profiles is kinesiophobia not an appropriate diagnosis?
3. Which measurement tool is used to assess kinesiophobia?
4. What is the primary consideration when approaching a patient with kinesiophobia?
5. Which attitudes and behaviors should be avoided when approaching a patient with kinesiophobia?
6. What are the factors contributing to the development of kinesiophobia?
7. What are appropriate home adaptation recommendations for patients with kinesiophobia?
8. Which behaviors should be avoided by patient relatives?
9. How do relatives’ attitudes affect the kinesiophobic condition?

The total duration of the intervention was five weeks, consisting of one class hour (50 min) per week. The control group received only the standard curriculum without any additional training.

### Statistical analysis

Data were analyzed using IBM SPSS Statistics 24 software. Descriptive statistics were presented as mean (X) and standard deviation (SD) for numerical variables and frequency and percentage for categorical variables (Tables 1 and 4). Differences in baseline demographic and academic characteristics between groups were analyzed using the chi-square test and independent samples t-test (Table 1).

**Table 1:** Comparison of descriptive characteristics and kinesiophobia knowledge levels of the groups

Variable/Category	Intervention Group (*n* = 46)	Control Group (*n* = 48)	Statistical Test	*p*
Gender (Female/Male)	38/8	41/7	*X*^2^ = 0.155	.694
Age (Mean ± SD)	21.69 ± 1.05	22.12 ± 2.56	*t* = −1.060	.292
GPA (Mean ± SD)	2.73 ± 0.35	2.50 ± 0.26	*t* = 3.593	.001*
Employment Status (Yes)	2 (%4.34)	6 (%12.5)	*X*^2^ = 1.954	.162
Voluntary Choice of Profession (Yes)	28 (%60.86)	29 (%60.41)	*X*^*2*^ = *0.002*	.965
Currently Likes Profession (Yes)	43 (%93.47)	41 (%85.41)	*X*^*2*^ = *1.637*	.201
Heard the Term KF (Yes)	23 (%50.0)	16 (%33.3)	*X*^2^ = 2.673	.102
Courses in which KF was heard (n)				
Kinesiology	8	0	-	-
Orthopedic Rehabilitation	3	2	-	-
Basic Measurement and Evaluation	1	1	-	-
Electrotherapy	0	2	-	-
Basic Exercise	0	2	-	-
Research Methods in Physiotherapy	1	0	-	-
Other	10	9	-	-
Knowledge of KF Concept (Yes)	22 (%47.8)	14 (%29.2)	*X*^2^ = 3.447	.063
Knowledge of Main Causes of KF (Yes)	4 (%8.69)	6 (%12.5)	*X*^*2*^ = *0.371*	.542
Knowledge of KF Scales (Yes)	1 (%2.2)	2 (%4.2)	*X*^2^ = 0.316	.574
Knowledge of KF Treatment Approaches (Yes)	0 (%0.0)	2 (%4.2)	*X*^2^ = 1.957	.162
Knowledge of Therapist Considerations in KF (Yes)	2 (%4,34)	4 (%8,33)	*X*^*2*^ = *0.613*	.434
Encounter with a Patient with KF (Yes)	5 (%10,86)	2 (%4,16)	*X*^*2*^ = *1.621*	.203

For the variables “Knowledge of KF Scales,” “Knowledge of KF Treatment Approaches,” “Knowledge of Therapeutic Considerations,” and “Encounter with a Patient with KF,” Fisher’s Exact Test results were used due to expected frequencies below 5. No comparison test was conducted for the “Courses in which KF was heard” variable because it included multiple responses

*KF* Kinesiophobia, *GPA* Grade Point Average, *FTR* Physiotherapy and Rehabilitation, *Mean* Mean value, *SD* Standard deviation, *n* Number of participants

χ^2^ indicates Pearson’s Chi-square test value. t indicates independent samples t-test value

*p* indicates statistical significance level (*p <.05 considered significant)

Changes in learning strategy scores over time within groups were analyzed using paired samples t-tests (Table 2). Between-group comparisons of pretest and posttest learning strategy scores were evaluated using independent samples t-tests (Table 3). Dominant learning strategies were categorized as visual, tactile, auditory, or kinesthetic and reported descriptively as frequencies and percentages before and after the intervention (Table 4). The effects of group and time on dominant learning strategies were analyzed using mixed ANOVA, and group × time interactions were examined (Table 5). Partial eta squared (η^2^p) effect sizes were reported for significant interactions.

**Table 2:** Changes in learning strategy domain over time in the ıntervention and control groups (within-group comparisons)

Group	Learning Style	Measurement	X	SD	D	t	df	*p*
	Visual	Pre-test	4,22	0,52	0,16	−5,781	45	** < 0.001**
		Post-test	4,38	0,45				
	Tactile	Pre-test	3,95	0,47	0,10	−3,969	45	** < 0.001**
Intervention Group		Post-test	4,05	0,49				
	Auditory	Pre-test	3,23	0,41	0,06	−2,558	45	**0.014**
		Post-test	3,29	0,40				
	Kinesthetic	Pre-test	3,11	0,43	0,16	−2,704	45	**0,010**
		Post-test	3,27	0,44				
	Visual	Pre-test	4,09	0,36	0,06	0,972	47	0.336
		Post-test	4,03	0,51				
Control Group	Tactile	Pre-test	3,98	0,37	0,00	0.000	47	0.336
		Post-test	3,98	0,44				
	Auditory	Pre-test	3,22	0,45	0,07	−1,199	47	0.237
		Post-test	3,28	0,50				
	Kinesthetic	Pre-test	3,14	0,42	0,04	−0,669	47	0,507
		Post-test	3,16	0,45				

Paired Samples t-test was used in the analyses. Mean: Arithmetic mean; SD: Standard deviation; D: Mean difference (Post-test – Pre-test); t: t-test value; df: degrees of freedom; p: statistical significance level. The significance level was accepted as α = 0.05. Values of *p* < 0.05 were considered statistically significant

**Table 3:** Between-group comparisons of learning strategy sub-parameters before and after the ıntervention

	Intervention Group	Control Group	t	df	p
Pre-intervention					
• Visual	4,22	4,09	1.362	92	0.177
• Tactile	3,95	3,98	−0,350	92	0.726
• Auditory	3,23	3,22	0.135	92	0.893
• Kinesthetic	3,11	3,14	−1,346	92	0.182
Post-intervention					
• Visual	4,38	4,03	3.462	92	0.001
• Tactile	4,05	3,98	0.643	92	0.522
• Auditory	3,29	3,28	0.019	92	1.000
• Kinesthetic	3,27	3.16	2.154	92	0.034

Data are presented as group means. Between-group comparisons were performed using the independent samples t-test. A value of *p* < 0.05 was considered statistically significant. *Mean* mean value, *SD* Standard deviation, *df* Degrees of freedom

**Table 4:** Distribution of dominant learning strategies before and after the ıntervention in the ıntervention and control groups

Learning Strategy	Group	Pre-test (n)	Pre-test (%)	Post-test (n)	Post-test (%)
Visual	Intervention Group	33	71,7	36	78,3
	Control Group	33	66,0	33	68,0
Tactile	Intervention Group	11	23,9	9	19,6
	Control Group	13	28,0	12	26,0
Auditory	Intervention Group	1	2,2	0	0,0
	Control Group	2	4,0	0	0,0
Kinesthetic	Intervention Group	1	2,2	1	2,2
	Control Group	0	0,0	2	4,0

Data are presented as frequency (n) and percentage (%). Dominant learning strategies were evaluated descriptively

**Table 5:** Comparison of changes in dominant learning strategy scores over time between groups (mixed measures ANOVA results)

Learning Style	Effect	Sum of Squares	df	Mean Square	F	p	n^2^_p_
Visual	Group × Time	0,277	1	0,277	14,834	**< 0,001**	0,118
Tactile	Group × Time	0,172	1	0,172	8,822	**0,004**	0,074
Auditory	Group × Time	0,265	1	0,265	32,852	**< 0,001**	0,230
Kinesthetic	Group × Time	0,000	1	0,000	0,000	1,000	0,000

A Mixed Measures ANOVA was conducted to compare changes in learning strategy scores over time between the intervention and control groups. F: F-test value; df: degrees of freedom; p: significance level; η^2^p: partial eta squared (effect size). The level of significance was accepted as α = 0.05

Changes in subscale and total scores of the Attitude Scale towards the Physiotherapy Profession before and after the intervention were evaluated using paired samples t-tests (Table 6). The level of statistical significance was set at α = 0.05, and *p* < 0.05 was considered statistically significant.

**Table 6:** Analysis of the within-group changes after training in the attitude scale toward the physiotherapy profession

**Scale/Subdimension**	**Grup ve Ölçüm**	**ORT ± SS**	t	P
Professional Satisfaction	Study Group Pre-test	35.34 ± 6.67	1.635	0.109
	Study Group Post-test	35.19 ± 6.65		
	Control Group Pre-test	40.29 ± 14.14	−1.552	0.127
	Control Group Post-test	41.83 ± 14.28		
Qualifications Required by the Profession	Study Group Pre-test	15.60 ± 3.10	1.354	0.183
	Study Group Post-test	15.54 ± 3.11		
	Control Group Pre-test	17.87 ± 6.69	−1.748	0.087
	Control Group Post-test	18.64 ± 6.95		
General Concerns About the Profession	Study Group Pre-test	14.71 ± 3.12	0.927	0.359
	Study Group Post-test	14.72 ± 3.14		
	Control Group Pre-test	15.95 ± 4.81	−0.855	0.397
	Control Group Post-test	16.16 ± 4.80		
Total Score	Study Group Pre-test	65.67 ± 9.79	1.701	0.096
	Study Group Post-test	65.45 ± 9.78		
	Control Group Pre-test	74.12 ± 24.24	−1.921	0.061
	Control Group Post-test	76.64 ± 24.30		

Paired Samples t-Test was used for within-group pre-test and post-test comparisons. Mean: Mean; SD: Standard Deviation. The level of significance was accepted as α = 0.05

## Results

### Demographic characteristics of participants and knowledge levels related to kinesiophobia

A total of 94 participants were included in the study, with 46 in the intervention group and 48 in the control group. The proportion of female participants was high in both groups, accounting for 82.6% in the intervention group and 85.4% in the control group. Male participants constituted 17.4% and 14.6% of the intervention and control groups, respectively. The mean age of participants was 21.69 ± 1.05 years in the intervention group and 22.12 ± 2.56 years in the control group. The intervention group had a significantly higher grade point average than the control group (2.73 ± 0.35 vs. 2.50 ± 0.26, *p* = 0.001). No other significant differences were observed between groups at baseline (Table 1).

Approximately 60% of participants reported that they had chosen the profession willingly, and the majority (85–93%) stated that they liked their profession. Awareness of the term kinesiophobia was reported by 50% of participants in the intervention group and 33.3% in the control group. The proportion of participants who reported knowing about kinesiophobia and its basic causes was low in both groups (8.7–12.5%). Knowledge regarding kinesiophobia assessment scales and treatment approaches was also limited in both groups. Finally, the rate of encountering patients with kinesiophobia was reported as 10.9% in the intervention group and 4.2% in the control group (Table 1).

The results of analyses evaluating within-group changes in learning strategy scores before and after the intervention in both groups are presented in Table 2. Compared with pretest measurements, significant increases were observed in all learning strategies in the intervention group following the game-based learning program. A significant increase was found in visual learning strategy scores (*p* < 0.001), and a similar statistically significant increase was observed in tactile learning style scores (*p* < 0.001). Additionally, significant increases were found in auditory and kinesthetic learning styles after the intervention (*p* < 0.05) (Table 2).

In the control group, no statistically significant changes were found in visual, tactile, auditory, or kinesthetic learning strategies between pretest and posttest measurements (*p* > 0.05) (Table 2).

Before the intervention, there were no statistically significant differences between the intervention and control groups in terms of learning strategy subparameters: visual (*p* = 0.177), tactile (*p* = 0.726), auditory (*p* = 0.893), and kinesthetic (*p* = 0.182), indicating similar baseline levels between groups (Table 3).

Post-intervention comparisons between groups showed that visual learning strategy scores were significantly higher in the intervention group than in the control group (*p* = 0.001). In addition, kinesthetic learning strategy scores differed significantly between groups, with higher scores in the intervention group compared to the control group (*p* = 0.034). No statistically significant differences were observed between groups in tactile (*p* = 0.522) or auditory (*p* = 1.000) learning strategy scores during the post-intervention assessment.

The distribution of dominant learning strategies in the intervention and control groups before and after the intervention is presented in Table 4. In both groups, visual learning strategy was the most frequently observed dominant learning strategy, followed by tactile learning strategy. No notable changes were observed in the distribution of dominant learning strategies after the intervention in either group.

Changes in learning strategy scores over time between the intervention and control groups were analyzed using mixed ANOVA (Table 5). Significant group × time interactions were observed for visual (F = 14.834, *p* < 0.001, η^2^p = 0.118), tactile (F = 8.822, *p* = 0.004, η^2^p = 0.074), and auditory (F = 32.852, *p* < 0.001, η^2^p = 0.230) learning strategy domains. No significant group × time interaction was identified for the kinesthetic domain (F = 0.000, *p* = 1.000, η^2^p = 0.000), indicating that changes over time did not differ between groups.

Pretest and posttest scores of the Attitude Scale towards the Physiotherapy Profession and its subdimensions were compared within the intervention and control groups (Table 6). According to the analysis results, no statistically significant changes were observed over time in professional satisfaction, professional qualifications, general concerns about the profession, or total attitude scores in either group (all *p* > 0.05).

## Discussion

This study investigated the effects of a game-based learning approach on physiotherapy students’ learning strategies and the time-dependent changes associated with this intervention. The findings indicate that the game-based learning intervention was associated with improvements in selected self-reported learning strategy domains, particularly visual, tactile, and auditory domains. These findings should be interpreted in the context of self-reported outcomes and the short-term nature of the intervention. These results align with previous research demonstrating that student-centered and active learning approaches enhance cognitive engagement, motivation, and learning outcomes [1, 2, 12]. In this respect, the present findings support the integration of game-based learning as an effective instructional strategy in physiotherapy education.

Analysis of baseline learning strategies showed that visual learning had the highest mean scores, while kinesthetic learning had the lowest. According to Kolb’s experiential learning theory [20], learning strategies are shaped by the interaction of concrete experience, reflective observation, abstract conceptualization, and active experimentation [45]. Contemporary educational models emphasize the importance of aligning teaching strategies with individual learning differences, particularly in health sciences education where continuous updating of knowledge and skills is required [24]. Game-based learning materials allow students to engage actively in the learning process, support peer interaction, and provide applied learning experiences that align with these educational demands. Although learning strategy frameworks have historically been used to describe individual learning preferences, contemporary evidence suggests that such preferences may be flexible and context-dependent rather than fixed learner characteristics [27, 32].

The observed improvements in visual and tactile learning strategies are consistent with existing literature highlighting the multi-sensory nature of game-based learning. Both digital and non-digital game-based environments facilitate information processing through multiple sensory channels, thereby enhancing learning retention [8, 16]. Studies involving health sciences students report that gamified and interactive teaching methods improve academic performance and promote meaningful learning experiences [14, 41]. The significant group × time interaction observed in auditory learning strategies further suggests that verbal explanations, peer discussion, and feedback during game-based activities play a crucial role in reinforcing learning.

Although kinesthetic learning scores increased significantly following the intervention, no significant group × time interaction was observed for dominant kinesthetic learning strategies. This finding suggests that short-term educational interventions may enhance kinesthetic awareness without fundamentally altering established learning preferences. Previous studies indicate that kinesthetic learning develops more strongly through prolonged experiential learning environments that emphasize motor skill acquisition and repetitive practice [25]. Therefore, the duration of the intervention may have been insufficient to produce measurable changes in dominant kinesthetic learning patterns.

Despite improvements in learning strategy scores, no substantial changes were observed in the distribution of dominant learning strategies between groups. These findings suggest that short-term educational interventions may influence learning strategy scores without substantially altering dominant learning preferences [27, 32]. Similar findings have been reported in health sciences education, where active and game-based learning approaches enhance engagement without producing rapid shifts in dominant learning preferences [35].

The intervention did not lead to statistically significant changes in students’ attitudes toward the physiotherapy profession. Professional attitudes are influenced by complex factors including clinical exposure, professional identity development, and long-term interaction with patients. Short-duration educational interventions may therefore be insufficient to elicit measurable attitudinal change. Prior research emphasizes that professional attitudes evolve gradually through clinical practice and experiential learning rather than isolated instructional methods [14, 21]. These findings suggest that while game-based learning effectively supports cognitive and sensory learning outcomes, its influence on professional attitudes should be evaluated over longer periods.

### Educational and clinical ımplications

The results demonstrate that game-based learning positively influences physiotherapy students’ learning strategies, particularly in visual, tactile, and auditory domains. The multi-sensory structure of game-based activities appears to activate multiple learning channels simultaneously, increasing engagement and supporting learning processes. Active learning approaches have been shown to enhance motivation and promote long-term knowledge retention in health sciences education [5, 41]. Incorporating game-based learning as a complementary method within physiotherapy curricula may therefore strengthen students’ learning processes.

The lack of change in dominant learning strategy distribution suggests that game-based learning reinforces existing learning tendencies rather than transforming individual preferences. Given the diversity of clinical contexts encountered by physiotherapy students, instructional approaches that support multiple learning strategies may support educational experiences relevant to clinical reasoning, problem-solving, and patient management [34]. Practice-based educational methods have been associated with enhanced flexibility in learning strategies and improved clinical performance [7, 33].

### Limitations and future research

A major strength of this study is its randomized pretest–posttest controlled design, which enabled evaluation of changes associated with the intervention. However, several limitations should be noted. The single-institution sample limits generalizability, the short intervention duration prevents conclusions about long-term effects, and reliance on self-report measures may introduce response bias. Additionally, the lack of objective performance outcomes restricts comprehensive evaluation. Future studies should include larger, multi-center samples and long-term follow-up to assess the sustainability of learning strategy changes. Investigating relationships between learning strategies and objective outcomes such as academic and clinical performance is recommended.

In addition, a significant baseline difference in grade point average was observed between groups, which may have influenced learning-related outcomes despite random allocation. The study relied exclusively on self-reported measures and did not include objective assessments of academic achievement, clinical reasoning, or practical performance. Furthermore, because participant blinding was not feasible and no active control condition was included, the potential influence of expectation and engagement effects cannot be excluded. Finally, the trial was retrospectively registered, which should be considered when interpreting the findings.

## Conclusion

This randomized controlled trial showed that a structured game-based learning approach was associated with improvements in selected self-reported learning strategy domains—particularly visual, tactile, and auditory domains—among final-year physiotherapy students. These findings highlight the capacity of game-based educational environments to support multi-sensory learning and enhance active cognitive engagement.

However, the intervention did not lead to short-term changes in dominant learning preferences or professional attitudes, suggesting that such stable constructs may require longer and more sustained educational exposure. Overall, game-based learning appears to be a valuable complementary teaching strategy in physiotherapy education, especially for supporting learning processes relevant to clinical reasoning. Future studies should investigate its long-term effects on clinical competence, decision-making, and professional development across diverse educational settings.

GBL may serve as a useful complementary educational approach for enhancing selected self-reported learning strategy domains in physiotherapy education. However, further studies incorporating objective educational outcomes and longer follow-up periods are needed to confirm its educational effectiveness.

## Supplementary Information

Supplementary Material 1.

## Data Availability

The data that support the findings of this study are not publicly available due to participant confidentiality and consent restrictions but are available from the corresponding author upon reasonable request.
