# Supplementary material for: Effects of a game-based learning ıntervention on self-reported learning strategies and professional attitudes in kinesiophobia management education: a parallel-group randomized controlled trial
Source: BMC Med Educ. 2026 Jun 25;26:1455. doi: 10.1186/s12909-026-09782-8 (PMC13563746; doi:10.1186/s12909-026-09782-8)
Supplement: Supplementary file 1 [file 12909_2026_9782_MOESM1_ESM.pdf]

CONSORT 2010 Checklist

Manuscript Title: Impact of Game-Based Learning on Learning Strategies and Professional Attitudes in Kinesiophobia Management Education: A Parallel-Group Randomized Controlled Trial

Section/Topic	Item No	Checklist Item	Reported on Page
Title and Abstract	1a	Identification as a randomized trial in the title	1
Title and Abstract	1b	Structured summary of trial design, methods, results, and conclusions	1–2
Introduction	2a	Scientific background and explanation of rationale	2–4
Introduction	2b	Specific objectives or hypotheses	5
Methods: Trial Design	3a	Description of trial design including allocation ratio	5
Methods: Trial Design	3b	Important changes to methods after trial commencement	Not applicable
Methods: Participants	4a	Eligibility criteria for participants	5–6
Methods: Participants	4b	Settings and locations where data were collected	5
Methods: Interventions	5	Detailed description of interventions for each group	7–8
Methods: Outcomes	6a	Defined primary and secondary outcome measures	6–7
Methods: Outcomes	6b	Changes to trial outcomes after commencement	Not applicable
Methods: Sample Size	7a	How sample size was determined	5
Methods: Sample Size	7b	Interim analyses and stopping guidelines	Not applicable
Methods: Randomisation	8a	Method used to generate random allocation sequence	6
Methods: Randomisation	8b	Type of randomisation and restrictions	6
Methods: Allocation Concealment	9	Mechanism used to implement allocation sequence	6
Methods: Implementation	10	Who generated allocation sequence, enrolled participants, and assigned interventions	6
Methods: Blinding	11a	Who was blinded after assignment to interventions	6
Methods: Blinding	11b	Similarity of interventions	Not applicable

Section/Topic	Item No	Checklist Item	Reported on Page
Methods: Statistical Methods	12a	Statistical methods used for primary and secondary outcomes	8–9
Methods: Statistical Methods	12b	Methods for additional analyses	8–9
Results: Participant Flow	13a	Numbers assigned, received treatment, analyzed	Figure 1, 9
Results: Participant Flow	13b	Losses and exclusions after randomisation	Figure 1, 9
Results: Recruitment	14a	Dates defining recruitment and follow-up periods	5
Results: Recruitment	14b	Why the trial ended or was stopped	Not applicable
Results: Baseline Data	15	Baseline demographic and clinical characteristics	Table 1
Results: Numbers Analysed	16	Number of participants included in each analysis	9
Results: Outcomes and Estimation	17a	Results for each outcome, effect size and precision	Tables 2–6
Results: Outcomes and Estimation	17b	Binary outcomes	Not applicable
Results: Ancillary Analyses	18	Results of subgroup or adjusted analyses	Table 5
Results: Harms	19	Important harms or unintended effects	No harms observed
Discussion: Limitations	20	Trial limitations	15
Discussion: Generalisability	21	Generalisability of findings	15
Discussion: Interpretation	22	Interpretation consistent with results	11–16
Other Information: Registration	23	Registration number and registry name	2, 6
Other Information: Protocol	24	Where full trial protocol can be accessed	Available from corresponding author upon request
Other Information: Funding	25	Sources of funding and role of funders	Funding section
